# Bedside Repair of Omphalocele

**Published:** 2012-10-01

**Authors:** Yavuz Yilmaz, Gulsum Kadioglu, Hulya Ozkan-Ulu, Sema Arayici, Omer Erdeve

**Affiliations:** Department of Pediatric Surgery, Zekai Tahir Burak Maternity Teaching Hospital, Ankara Turkey.; 1 Department of Neonatology, Zekai Tahir Burak Maternity Teaching Hospital, Ankara Turkey.

**Dear Sir**

An omphalocele is one of the developmental anomalies of the abdominal wall. Incidence is 1.5-3 case per 10000 births. The abdominal viscera are surrounded by the Wharton jelly, peritoneum and amnion and contained in a translucent sac. The sac protrudes in the mid-line, through the umbilicus. Omphalocele is associated with additional anomalies in about 50-70% of cases. After the birth, defect is treated according to the defect size and the surgeon's preference [1-3].

A female infant of 36-week-gestation (weight 2420 g) was born by elective C-section to a 27-year-old gravida 1 mother. APGAR scores were 7 and 9 at first and fifth minutes, respectively. General physical examination showed an omphalocele sac approximately 5x5cm in size containing intestinal loops (Fig. 1), other systemic and cardiac assessments were normal. Sac was wrapped with a sterile wet gauze pad. The patient underwent orogastric decompression. At the postnatal forth hour, a standing abdominal X-ray was taken, that was non suggestive. With 1 ml/min of oxygen support, we covered the operative area under the terms of surgical sterilization procedures at the bedside. The mid-gut in the omphalocele sac was pushed into the abdomen easily. During the repair process patient's respiratory and cardiac data were monitored. When intestine was reduced, prilocaine 0.5 ml was injected to the junction line of omphalocele sac and skin. An incision was given at the junction line with sac and skin. After removal of the sac, the skin was repaired with a continuous 4-0 polyglactin suture, keeping the fascia intact (Fig. 2). Incision was closed with wound dressing. The process took about 5 minutes. Oral feeding was commenced 2 hours after the operation. The patient was discharged from hospital at 5th postoperative day. Patient is on follow up.

**Figure:**
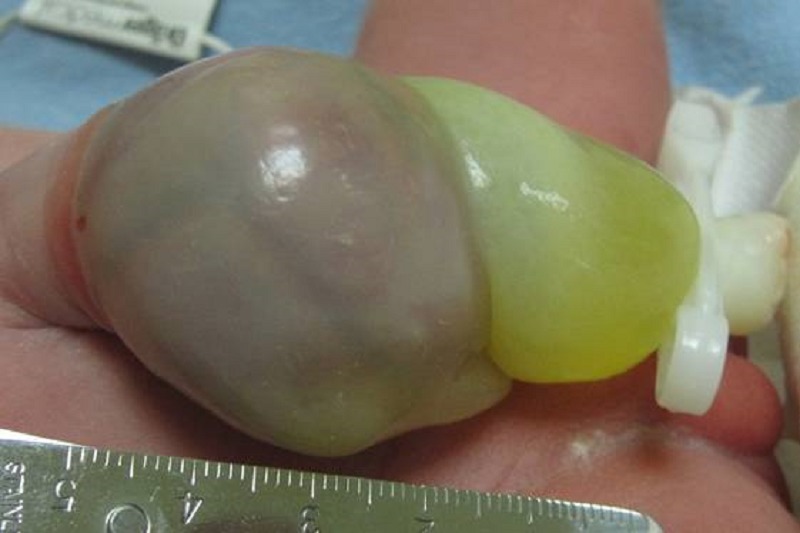
Figure 1: Omphalocele sac.

**Figure:**
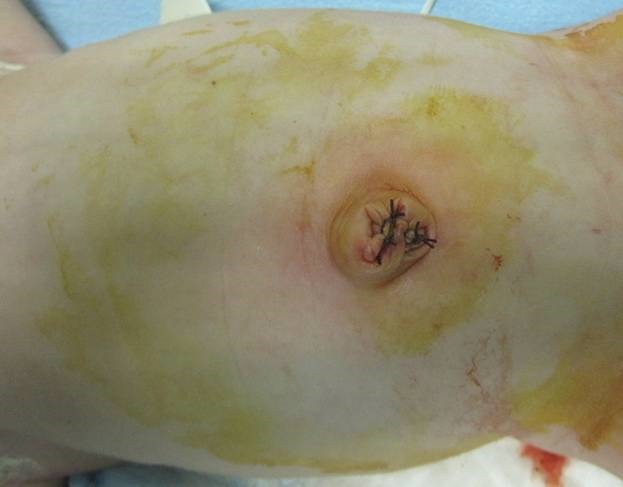
Figure 2: After bedside closure.

Omphalocele which is less than 4 cm in size is suitable for primary repair; whereas, defects over 5 cm are likely to contain liver and thus intra-abdominal volume may be insufficient after reduction of the intestines to the abdomen [4,5]. Compartment syndrome due to increased intra-abdominal pressure is the most serious complication after the difficult reductions. Dunn and Fonkalsrud reported that a 12% incidence of complications of increased intra-abdominal pressure after closure, including acute hepatic congestion, renal failure, and bowel infarction [5,6]. In patients with large defects, the primary repair is not possible and silo repair can be preferred. The other techniques are alloderm patch defect, vacuum-assisted closure, tissue expanders, using other types of mesh materials. The method of delayed closure is another technique for omphalocele closure [6,7]. EDMR-No GA (Elective delayed mid-gut reduction without anesthesia) method was applied in 1998 for the first time by Bianchi and Dickinson in the cases of gastroschisis [8,9]. 

The most important criteria for bedside reduction of omphalocele are patient's good general condition, small size defect, and systemic and cardiopulmonary well-being. The operation theatre must be kept in hand in case of failure of the reduction or other complications. We suggest that the method can be applied in selected cases because of the advantages like short duration, avoidance of general anesthesia, early recovery, and short hospital stay.

## Footnotes

**Source of Support:** Nil

**Conflict of Interest:** None
